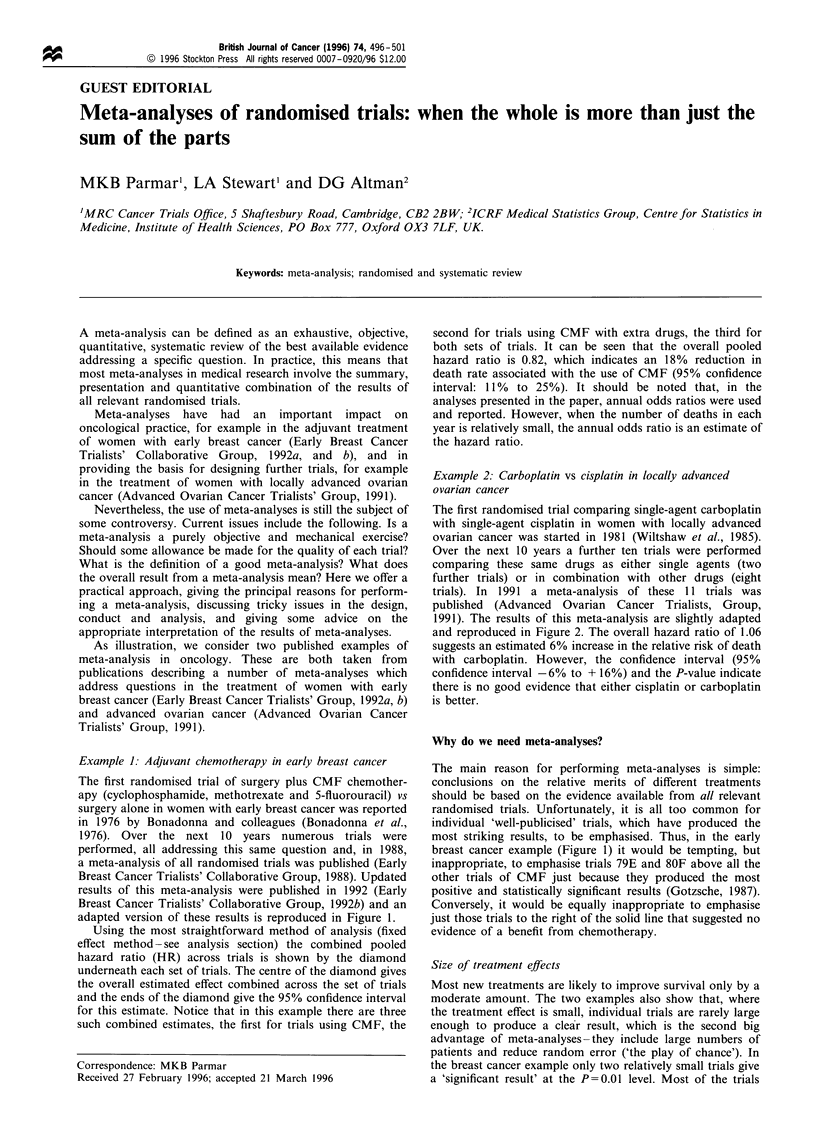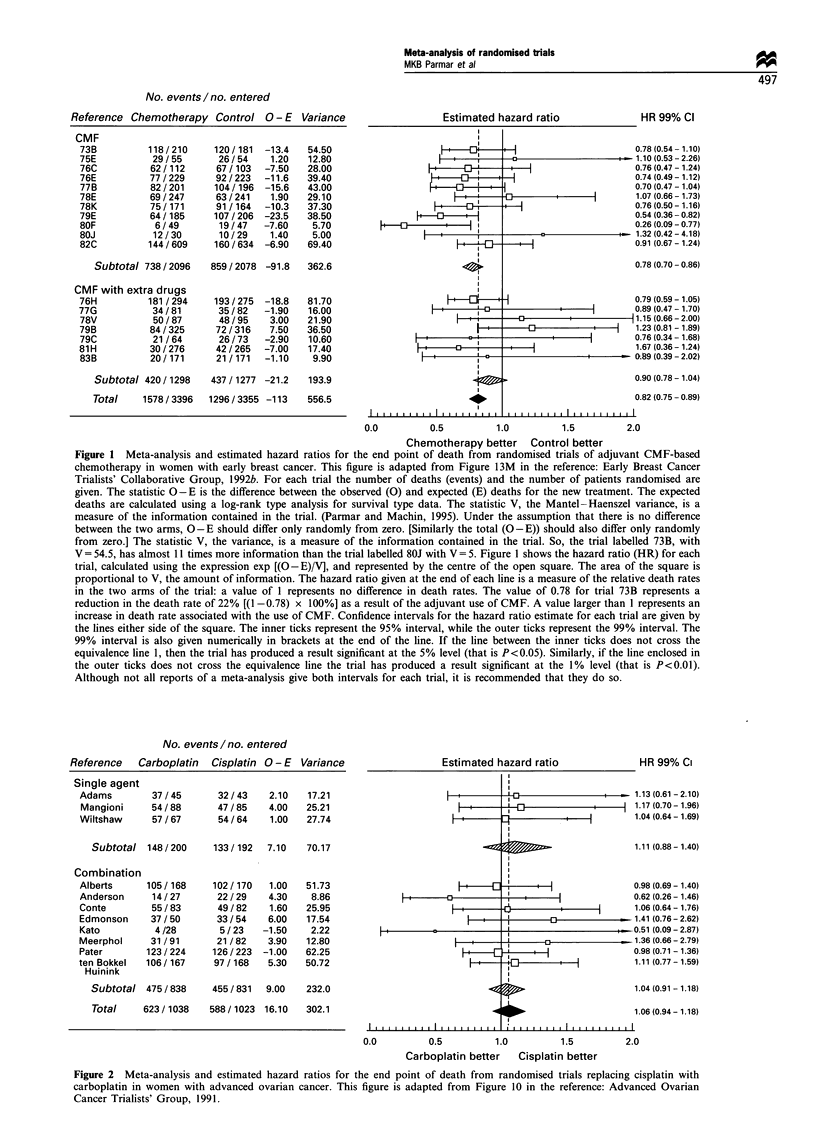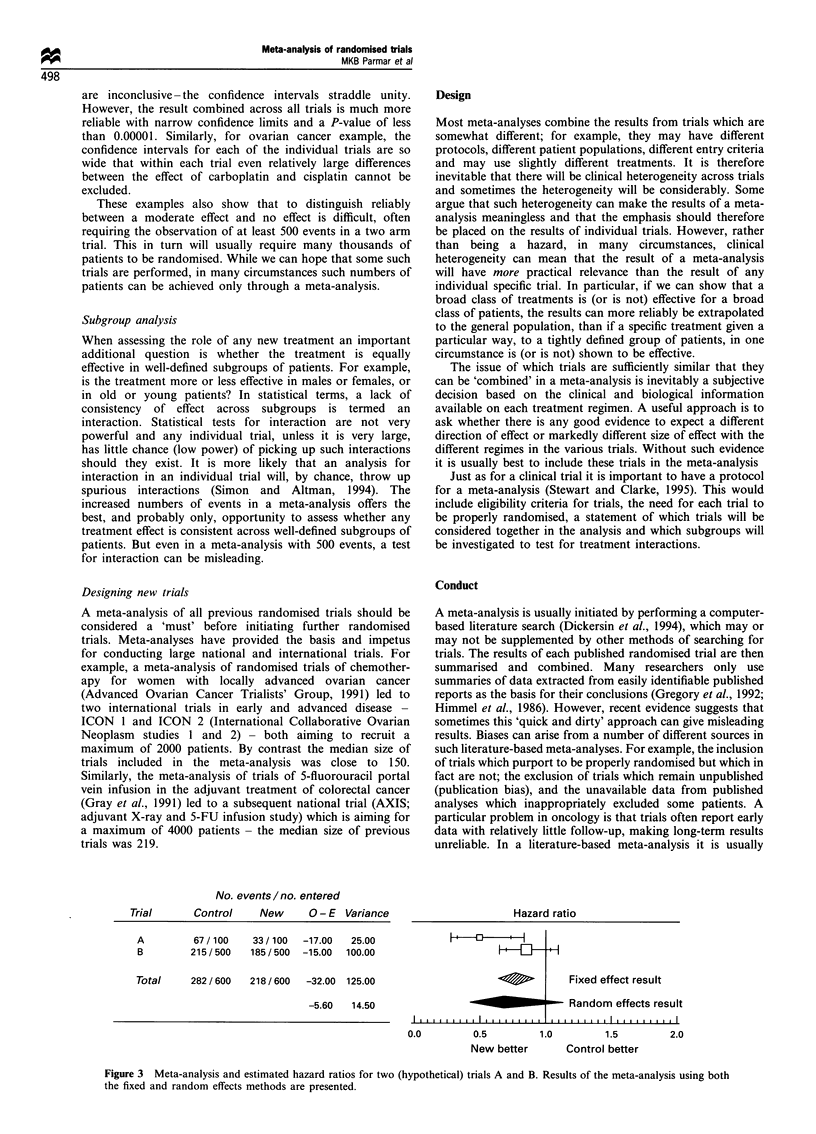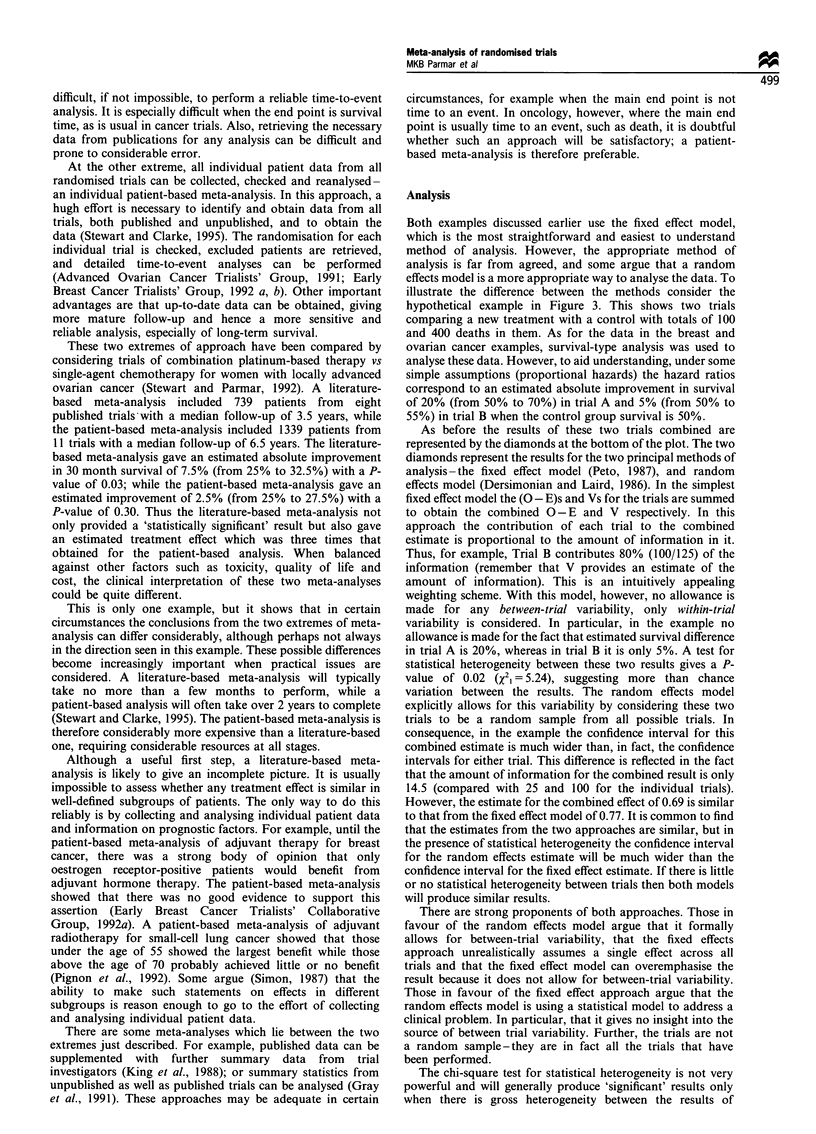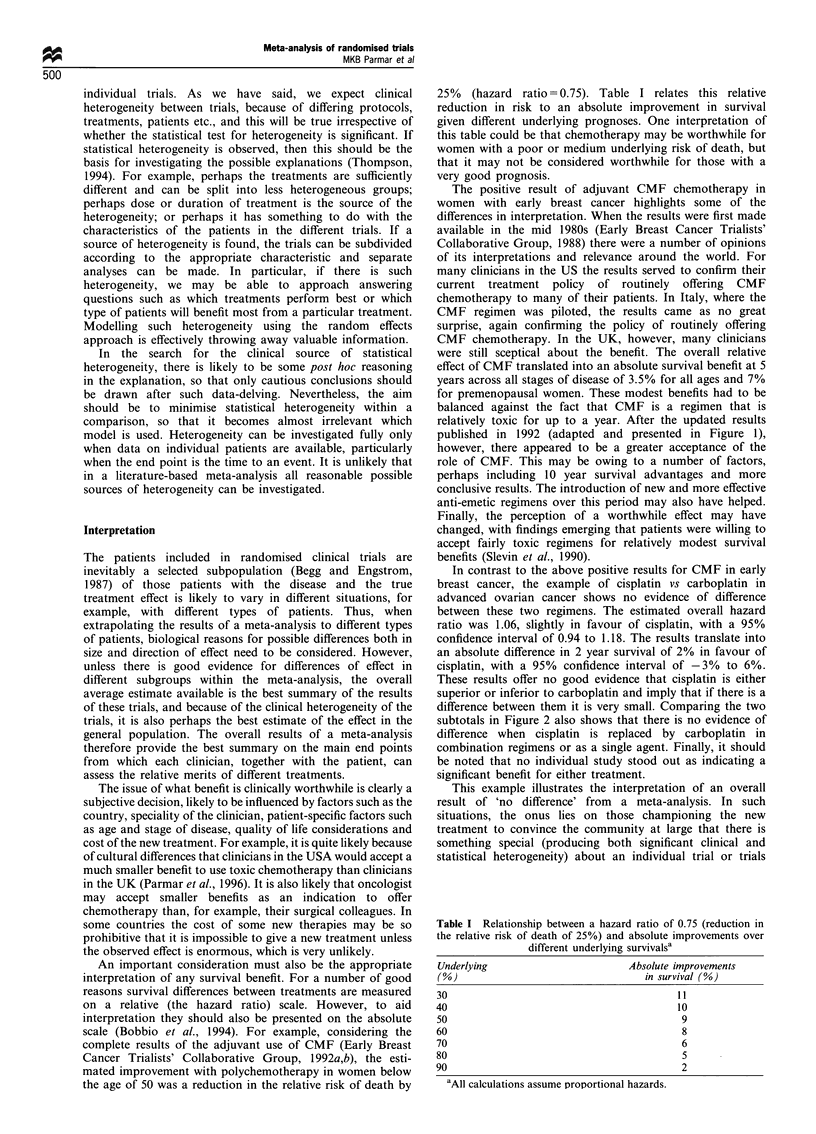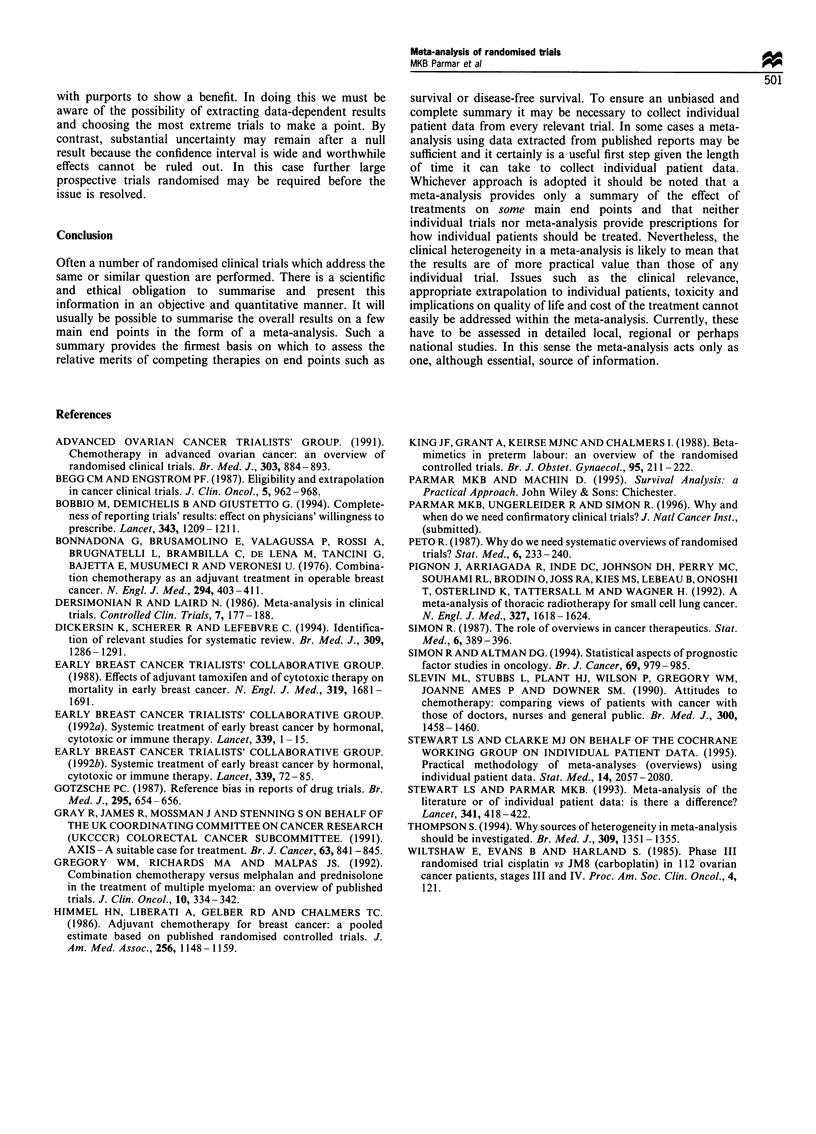# Meta-analyses of randomised trials: when the whole is more than just the sum of the parts.

**DOI:** 10.1038/bjc.1996.392

**Published:** 1996-08

**Authors:** M. K. Parmar, L. A. Stewart, D. G. Altman


British Journal of Cancer (1996) 74, 496-501
© 1996 Stockton Press All rights reserved 0007-0920/96 $12.00

GUEST EDITORIAL

### Meta-analyses of randomised trials: when the whole is more than just the sum of the parts

MKB Parmar[1], LA Stewart[1] and DG Altman[2]

[1]*MRC Cancer Trials Office, 5 Shaftesbury Road, Cambridge, CB2 2BW;* [2]*ICRF Medical Statistics Group, Centre for Statistics in Medicine, Institute of Health Sciences, PO Box 777, Oxford OX3 7LF, UK.*

**Keywords:** meta-analysis; randomised and systematic review

A meta-analysis can be defined as an exhaustive, objective, quantitative, systematic review of the best available evidence addressing a specific question. In practice, this means that most meta-analyses in medical research involve the summary, presentation and quantitative combination of the results of all relevant randomised trials.

Meta-analyses have had an important impact on oncological practice, for example in the adjuvant treatment of women with early breast cancer (Early Breast Cancer Trialists' Collaborative Group, 1992*a*, and *b*), and in providing the basis for designing further trials, for example in the treatment of women with locally advanced ovarian cancer (Advanced Ovarian Cancer Trialists' Group, 1991).

Nevertheless, the use of meta-analyses is still the subject of some controversy. Current issues include the following. Is a meta-analysis a purely objective and mechanical exercise? Should some allowance be made for the quality of each trial? What is the definition of a good meta-analysis? What does the overall result from a meta-analysis mean? Here we offer a practical approach, giving the principal reasons for performing a meta-analysis, discussing tricky issues in the design, conduct and analysis, and giving some advice on the appropriate interpretation of the results of meta-analyses.

As illustration, we consider two published examples of meta-analysis in oncology. These are both taken from publications describing a number of meta-analyses which address questions in the treatment of women with early breast cancer (Early Breast Cancer Trialists' Group, 1992*a*, *b*) and advanced ovarian cancer (Advanced Ovarian Cancer Trialists' Group, 1991).

*Example 1: Adjuvant chemotherapy in early breast cancer*

The first randomised trial of surgery plus CMF chemotherapy (cyclophosphamide, methotrexate and 5-fluorouracil) *vs* surgery alone in women with early breast cancer was reported in 1976 by Bonadonna and colleagues (Bonadonna *et al.*, 1976). Over the next 10 years numerous trials were performed, all addressing this same question and, in 1988, a meta-analysis of all randomised trials was published (Early Breast Cancer Trialists' Collaborative Group, 1988). Updated results of this meta-analysis were published in 1992 (Early Breast Cancer Trialists' Collaborative Group, 1992*b*) and an adapted version of these results is reproduced in Figure 1.

Using the most straightforward method of analysis (fixed effect method – see analysis section) the combined pooled hazard ratio (HR) across trials is shown by the diamond underneath each set of trials. The centre of the diamond gives the overall estimated effect combined across the set of trials and the ends of the diamond give the 95% confidence interval for this estimate. Notice that in this example there are three such combined estimates, the first for trials using CMF, the second for trials using CMF with extra drugs, the third for both sets of trials. It can be seen that the overall pooled hazard ratio is 0.82, which indicates an 18% reduction in death rate associated with the use of CMF (95% confidence interval: 11% to 25%). It should be noted that, in the analyses presented in the paper, annual odds ratios were used and reported. However, when the number of deaths in each year is relatively small, the annual odds ratio is an estimate of the hazard ratio.

*Example 2: Carboplatin* vs *cisplatin in locally advanced ovarian cancer*

The first randomised trial comparing single-agent carboplatin with single-agent cisplatin in women with locally advanced ovarian cancer was started in 1981 (Wiltshaw *et al.*, 1985). Over the next 10 years a further ten trials were performed comparing these same drugs as either single agents (two further trials) or in combination with other drugs (eight trials). In 1991 a meta-analysis of these 11 trials was published (Advanced Ovarian Cancer Trialists, Group, 1991). The results of this meta-analysis are slightly adapted and reproduced in Figure 2. The overall hazard ratio of 1.06 suggests an estimated 6% increase in the relative risk of death with carboplatin. However, the confidence interval (95% confidence interval −6% to +16%) and the *P*-value indicate there is no good evidence that either cisplatin or carboplatin is better.

### Why do we need meta-analyses?

The main reason for performing meta-analyses is simple: conclusions on the relative merits of different treatments should be based on the evidence available from *all* relevant randomised trials. Unfortunately, it is all too common for individual 'well-publicised' trials, which have produced the most striking results, to be emphasised. Thus, in the early breast cancer example (Figure 1) it would be tempting, but inappropriate, to emphasise trials 79E and 80F above all the other trials of CMF just because they produced the most positive and statistically significant results (Gotzsche, 1987). Conversely, it would be equally inappropriate to emphasise just those trials to the right of the solid line that suggested no evidence of a benefit from chemotherapy.

*Size of treatment effects*

Most new treatments are likely to improve survival only by a moderate amount. The two examples also show that, where the treatment effect is small, individual trials are rarely large enough to produce a clear result, which is the second big advantage of meta-analyses – they include large numbers of patients and reduce random error ('the play of chance'). In the breast cancer example only two relatively small trials give a 'significant result' at the $P=0.01$ level. Most of the trials

Correspondence: MKB Parmar
Received 27 February 1996; accepted 21 March 1996

| Reference | Chemotherapy (No. events / no. entered) | Control (No. events / no. entered) | O − E | Variance | HR 99% CI |
|---|---|---|---|---|---|
| **CMF** | | | | | |
| 73B | 118 / 210 | 120 / 181 | -13.4 | 54.50 | 0.78 (0.54 - 1.10) |
| 75E | 29 / 55 | 26 / 54 | 1.20 | 12.80 | 1.10 (0.53 - 2.26) |
| 76C | 62 / 112 | 67 / 103 | -7.50 | 28.00 | 0.76 (0.47 - 1.24) |
| 76E | 77 / 229 | 92 / 223 | -11.6 | 39.40 | 0.74 (0.49 - 1.12) |
| 77B | 82 / 201 | 104 / 196 | -15.6 | 43.00 | 0.70 (0.47 - 1.04) |
| 78E | 69 / 247 | 63 / 241 | 1.90 | 29.10 | 1.07 (0.66 - 1.73) |
| 78K | 75 / 171 | 91 / 164 | -10.3 | 37.30 | 0.76 (0.50 - 1.16) |
| 79E | 64 / 185 | 107 / 206 | -23.5 | 38.50 | 0.54 (0.36 - 0.82) |
| 80F | 6 / 49 | 19 / 47 | -7.60 | 5.70 | 0.26 (0.09 - 0.77) |
| 80J | 12 / 30 | 10 / 29 | 1.40 | 5.00 | 1.32 (0.42 - 4.18) |
| 82C | 144 / 609 | 160 / 634 | -6.90 | 69.40 | 0.91 (0.67 - 1.24) |
| *Subtotal* | 738 / 2096 | 859 / 2078 | -91.8 | 362.6 | 0.78 (0.70 - 0.86) |
| **CMF with extra drugs** | | | | | |
| 76H | 181 / 294 | 193 / 275 | -18.8 | 81.70 | 0.79 (0.59 - 1.05) |
| 77G | 34 / 81 | 35 / 82 | -1.90 | 16.00 | 0.89 (0.47 - 1.70) |
| 78V | 50 / 87 | 48 / 95 | 3.00 | 21.90 | 1.15 (0.66 - 2.00) |
| 79B | 84 / 325 | 72 / 316 | 7.50 | 36.50 | 1.23 (0.81 - 1.89) |
| 79C | 21 / 64 | 26 / 73 | -2.90 | 10.60 | 0.76 (0.34 - 1.68) |
| 81H | 30 / 276 | 42 / 265 | -7.00 | 17.40 | 1.67 (0.36 - 1.24) |
| 83B | 20 / 171 | 21 / 171 | -1.10 | 9.90 | 0.89 (0.39 - 2.02) |
| *Subtotal* | 420 / 1298 | 437 / 1277 | -21.2 | 193.9 | 0.90 (0.78 - 1.04) |
| *Total* | 1578 / 3396 | 1296 / 3355 | -113 | 556.5 | 0.82 (0.75 - 0.89) |

**Figure 1** Meta-analysis and estimated hazard ratios for the end point of death from randomised trials of adjuvant CMF-based chemotherapy in women with early breast cancer. This figure is adapted from Figure 13M in the reference: Early Breast Cancer Trialists' Collaborative Group, 1992*b*. For each trial the number of deaths (events) and the number of patients randomised are given. The statistic O − E is the difference between the observed (O) and expected (E) deaths for the new treatment. The expected deaths are calculated using a log-rank type analysis for survival type data. The statistic V, the Mantel−Haenszel variance, is a measure of the information contained in the trial. (Parmar and Machin, 1995). Under the assumption that there is no difference between the two arms, O − E should differ only randomly from zero. [Similarly the total (O − E)) should also differ only randomly from zero.] The statistic V, the variance, is a measure of the information contained in the trial. So, the trial labelled 73B, with V = 54.5, has almost 11 times more information than the trial labelled 80J with V = 5. Figure 1 shows the hazard ratio (HR) for each trial, calculated using the expression exp [(O − E)/V], and represented by the centre of the open square. The area of the square is proportional to V, the amount of information. The hazard ratio given at the end of each line is a measure of the relative death rates in the two arms of the trial: a value of 1 represents no difference in death rates. The value of 0.78 for trial 73B represents a reduction in the death rate of 22% [(1 − 0.78) × 100%] as a result of the adjuvant use of CMF. A value larger than 1 represents an increase in death rate associated with the use of CMF. Confidence intervals for the hazard ratio estimate for each trial are given by the lines either side of the square. The inner ticks represent the 95% interval, while the outer ticks represent the 99% interval. The 99% interval is also given numerically in brackets at the end of the line. If the line between the inner ticks does not cross the equivalence line 1, then the trial has produced a result significant at the 5% level (that is $P < 0.05$). Similarly, if the line enclosed in the outer ticks does not cross the equivalence line the trial has produced a result significant at the 1% level (that is $P < 0.01$). Although not all reports of a meta-analysis give both intervals for each trial, it is recommended that they do so.

| Reference | Carboplatin (No. events / no. entered) | Cisplatin (No. events / no. entered) | O − E | Variance | HR 99% CI |
|---|---|---|---|---|---|
| **Single agent** | | | | | |
| Adams | 37 / 45 | 32 / 43 | 2.10 | 17.21 | 1.13 (0.61 - 2.10) |
| Mangioni | 54 / 88 | 47 / 85 | 4.00 | 25.21 | 1.17 (0.70 - 1.96) |
| Wiltshaw | 57 / 67 | 54 / 64 | 1.00 | 27.74 | 1.04 (0.64 - 1.69) |
| *Subtotal* | 148 / 200 | 133 / 192 | 7.10 | 70.17 | 1.11 (0.88 - 1.40) |
| **Combination** | | | | | |
| Alberts | 105 / 168 | 102 / 170 | 1.00 | 51.73 | 0.98 (0.69 - 1.40) |
| Anderson | 14 / 27 | 22 / 29 | 4.30 | 8.86 | 0.62 (0.26 - 1.46) |
| Conte | 55 / 83 | 49 / 82 | 1.60 | 25.95 | 1.06 (0.64 - 1.76) |
| Edmonson | 37 / 50 | 33 / 54 | 6.00 | 17.54 | 1.41 (0.76 - 2.62) |
| Kato | 4 /28 | 5 / 23 | -1.50 | 2.22 | 0.51 (0.09 - 2.87) |
| Meerphol | 31 / 91 | 21 / 82 | 3.90 | 12.80 | 1.36 (0.66 - 2.79) |
| Pater | 123 / 224 | 126 / 223 | -1.00 | 62.25 | 0.98 (0.71 - 1.36) |
| ten Bokkel Huinink | 106 / 167 | 97 / 168 | 5.30 | 50.72 | 1.11 (0.77 - 1.59) |
| *Subtotal* | 475 / 838 | 455 / 831 | 9.00 | 232.0 | 1.04 (0.91 - 1.18) |
| *Total* | 623 / 1038 | 588 / 1023 | 16.10 | 302.1 | 1.06 (0.94 - 1.18) |

**Figure 2** Meta-analysis and estimated hazard ratios for the end point of death from randomised trials replacing cisplatin with carboplatin in women with advanced ovarian cancer. This figure is adapted from Figure 10 in the reference: Advanced Ovarian Cancer Trialists' Group, 1991.

are inconclusive – the confidence intervals straddle unity. However, the result combined across all trials is much more reliable with narrow confidence limits and a *P*-value of less than 0.00001. Similarly, for ovarian cancer example, the confidence intervals for each of the individual trials are so wide that within each trial even relatively large differences between the effect of carboplatin and cisplatin cannot be excluded.

These examples also show that to distinguish reliably between a moderate effect and no effect is difficult, often requiring the observation of at least 500 events in a two arm trial. This in turn will usually require many thousands of patients to be randomised. While we can hope that some such trials are performed, in many circumstances such numbers of patients can be achieved only through a meta-analysis.

### *Subgroup analysis*

When assessing the role of any new treatment an important additional question is whether the treatment is equally effective in well-defined subgroups of patients. For example, is the treatment more or less effective in males or females, or in old or young patients? In statistical terms, a lack of consistency of effect across subgroups is termed an interaction. Statistical tests for interaction are not very powerful and any individual trial, unless it is very large, has little chance (low power) of picking up such interactions should they exist. It is more likely that an analysis for interaction in an individual trial will, by chance, throw up spurious interactions (Simon and Altman, 1994). The increased numbers of events in a meta-analysis offers the best, and probably only, opportunity to assess whether any treatment effect is consistent across well-defined subgroups of patients. But even in a meta-analysis with 500 events, a test for interaction can be misleading.

### *Designing new trials*

A meta-analysis of all previous randomised trials should be considered a 'must' before initiating further randomised trials. Meta-analyses have provided the basis and impetus for conducting large national and international trials. For example, a meta-analysis of randomised trials of chemotherapy for women with locally advanced ovarian cancer (Advanced Ovarian Cancer Trialists' Group, 1991) led to two international trials in early and advanced disease – ICON 1 and ICON 2 (International Collaborative Ovarian Neoplasm studies 1 and 2) – both aiming to recruit a maximum of 2000 patients. By contrast the median size of trials included in the meta-analysis was close to 150. Similarly, the meta-analysis of trials of 5-fluorouracil portal vein infusion in the adjuvant treatment of colorectal cancer (Gray *et al.*, 1991) led to a subsequent national trial (AXIS; adjuvant X-ray and 5-FU infusion study) which is aiming for a maximum of 4000 patients – the median size of previous trials was 219.

### Design

Most meta-analyses combine the results from trials which are somewhat different; for example, they may have different protocols, different patient populations, different entry criteria and may use slightly different treatments. It is therefore inevitable that there will be clinical heterogeneity across trials and sometimes the heterogeneity will be considerably. Some argue that such heterogeneity can make the results of a meta-analysis meaningless and that the emphasis should therefore be placed on the results of individual trials. However, rather than being a hazard, in many circumstances, clinical heterogeneity can mean that the result of a meta-analysis will have *more* practical relevance than the result of any individual specific trial. In particular, if we can show that a broad class of treatments is (or is not) effective for a broad class of patients, the results can more reliably be extrapolated to the general population, than if a specific treatment given a particular way, to a tightly defined group of patients, in one circumstance is (or is not) shown to be effective.

The issue of which trials are sufficiently similar that they can be 'combined' in a meta-analysis is inevitably a subjective decision based on the clinical and biological information available on each treatment regimen. A useful approach is to ask whether there is any good evidence to expect a different direction of effect or markedly different size of effect with the different regimes in the various trials. Without such evidence it is usually best to include these trials in the meta-analysis

Just as for a clinical trial it is important to have a protocol for a meta-analysis (Stewart and Clarke, 1995). This would include eligibility criteria for trials, the need for each trial to be properly randomised, a statement of which trials will be considered together in the analysis and which subgroups will be investigated to test for treatment interactions.

### Conduct

A meta-analysis is usually initiated by performing a computer-based literature search (Dickersin *et al.*, 1994), which may or may not be supplemented by other methods of searching for trials. The results of each published randomised trial are then summarised and combined. Many researchers only use summaries of data extracted from easily identifiable published reports as the basis for their conclusions (Gregory *et al.*, 1992; Himmel *et al.*, 1986). However, recent evidence suggests that sometimes this 'quick and dirty' approach can give misleading results. Biases can arise from a number of different sources in such literature-based meta-analyses. For example, the inclusion of trials which purport to be properly randomised but which in fact are not; the exclusion of trials which remain unpublished (publication bias), and the unavailable data from published analyses which inappropriately excluded some patients. A particular problem in oncology is that trials often report early data with relatively little follow-up, making long-term results unreliable. In a literature-based meta-analysis it is usually

| | No. events / no. entered | | | |
|---|---|---|---|---|
| *Trial* | *Control* | *New* | *O – E* | *Variance* |
| A | 67 / 100 | 33 / 100 | -17.00 | 25.00 |
| B | 215 / 500 | 185 / 500 | -15.00 | 100.00 |
| *Total* | 282 / 600 | 218 / 600 | -32.00 | 125.00 |
| | | | -5.60 | 14.50 |

**Figure 3** Meta-analysis and estimated hazard ratios for two (hypothetical) trials A and B. Results of the meta-analysis using both the fixed and random effects methods are presented.

difficult, if not impossible, to perform a reliable time-to-event analysis. It is especially difficult when the end point is survival time, as is usual in cancer trials. Also, retrieving the necessary data from publications for any analysis can be difficult and prone to considerable error.

At the other extreme, all individual patient data from all randomised trials can be collected, checked and reanalysed – an individual patient-based meta-analysis. In this approach, a hugh effort is necessary to identify and obtain data from all trials, both published and unpublished, and to obtain the data (Stewart and Clarke, 1995). The randomisation for each individual trial is checked, excluded patients are retrieved, and detailed time-to-event analyses can be performed (Advanced Ovarian Cancer Trialists' Group, 1991; Early Breast Cancer Trialists' Group, 1992 *a*, *b*). Other important advantages are that up-to-date data can be obtained, giving more mature follow-up and hence a more sensitive and reliable analysis, especially of long-term survival.

These two extremes of approach have been compared by considering trials of combination platinum-based therapy *vs* single-agent chemotherapy for women with locally advanced ovarian cancer (Stewart and Parmar, 1992). A literature-based meta-analysis included 739 patients from eight published trials with a median follow-up of 3.5 years, while the patient-based meta-analysis included 1339 patients from 11 trials with a median follow-up of 6.5 years. The literature-based meta-analysis gave an estimated absolute improvement in 30 month survival of 7.5% (from 25% to 32.5%) with a *P*-value of 0.03; while the patient-based meta-analysis gave an estimated improvement of 2.5% (from 25% to 27.5%) with a *P*-value of 0.30. Thus the literature-based meta-analysis not only provided a 'statistically significant' result but also gave an estimated treatment effect which was three times that obtained for the patient-based analysis. When balanced against other factors such as toxicity, quality of life and cost, the clinical interpretation of these two meta-analyses could be quite different.

This is only one example, but it shows that in certain circumstances the conclusions from the two extremes of meta-analysis can differ considerably, although perhaps not always in the direction seen in this example. These possible differences become increasingly important when practical issues are considered. A literature-based meta-analysis will typically take no more than a few months to perform, while a patient-based analysis will often take over 2 years to complete (Stewart and Clarke, 1995). The patient-based meta-analysis is therefore considerably more expensive than a literature-based one, requiring considerable resources at all stages.

Although a useful first step, a literature-based meta-analysis is likely to give an incomplete picture. It is usually impossible to assess whether any treatment effect is similar in well-defined subgroups of patients. The only way to do this reliably is by collecting and analysing individual patient data and information on prognostic factors. For example, until the patient-based meta-analysis of adjuvant therapy for breast cancer, there was a strong body of opinion that only oestrogen receptor-positive patients would benefit from adjuvant hormone therapy. The patient-based meta-analysis showed that there was no good evidence to support this assertion (Early Breast Cancer Trialists' Collaborative Group, 1992*a*). A patient-based meta-analysis of adjuvant radiotherapy for small-cell lung cancer showed that those under the age of 55 showed the largest benefit while those above the age of 70 probably achieved little or no benefit (Pignon *et al.*, 1992). Some argue (Simon, 1987) that the ability to make such statements on effects in different subgroups is reason enough to go to the effort of collecting and analysing individual patient data.

There are some meta-analyses which lie between the two extremes just described. For example, published data can be supplemented with further summary data from trial investigators (King *et al.*, 1988); or summary statistics from unpublished as well as published trials can be analysed (Gray *et al.*, 1991). These approaches may be adequate in certain circumstances, for example when the main end point is not time to an event. In oncology, however, where the main end point is usually time to an event, such as death, it is doubtful whether such an approach will be satisfactory; a patient-based meta-analysis is therefore preferable.

### Analysis

Both examples discussed earlier use the fixed effect model, which is the most straightforward and easiest to understand method of analysis. However, the appropriate method of analysis is far from agreed, and some argue that a random effects model is a more appropriate way to analyse the data. To illustrate the difference between the methods consider the hypothetical example in Figure 3. This shows two trials comparing a new treatment with a control with totals of 100 and 400 deaths in them. As for the data in the breast and ovarian cancer examples, survival-type analysis was used to analyse these data. However, to aid understanding, under some simple assumptions (proportional hazards) the hazard ratios correspond to an estimated absolute improvement in survival of 20% (from 50% to 70%) in trial A and 5% (from 50% to 55%) in trial B when the control group survival is 50%.

As before the results of these two trials combined are represented by the diamonds at the bottom of the plot. The two diamonds represent the results for the two principal methods of analysis – the fixed effect model (Peto, 1987), and random effects model (Dersimonian and Laird, 1986). In the simplest fixed effect model the (O – E)s and Vs for the trials are summed to obtain the combined O – E and V respectively. In this approach the contribution of each trial to the combined estimate is proportional to the amount of information in it. Thus, for example, Trial B contributes 80% (100/125) of the information (remember that V provides an estimate of the amount of information). This is an intuitively appealing weighting scheme. With this model, however, no allowance is made for any *between-trial* variability, only *within-trial* variability is considered. In particular, in the example no allowance is made for the fact that estimated survival difference in trial A is 20%, whereas in trial B it is only 5%. A test for statistical heterogeneity between these two results gives a *P*-value of 0.02 ($\chi^2_1 = 5.24$), suggesting more than chance variation between the results. The random effects model explicitly allows for this variability by considering these two trials to be a random sample from all possible trials. In consequence, in the example the confidence interval for this combined estimate is much wider than, in fact, the confidence intervals for either trial. This difference is reflected in the fact that the amount of information for the combined result is only 14.5 (compared with 25 and 100 for the individual trials). However, the estimate for the combined effect of 0.69 is similar to that from the fixed effect model of 0.77. It is common to find that the estimates from the two approaches are similar, but in the presence of statistical heterogeneity the confidence interval for the random effects estimate will be much wider than the confidence interval for the fixed effect estimate. If there is little or no statistical heterogeneity between trials then both models will produce similar results.

There are strong proponents of both approaches. Those in favour of the random effects model argue that it formally allows for between-trial variability, that the fixed effects approach unrealistically assumes a single effect across all trials and that the fixed effect model can overemphasise the result because it does not allow for between-trial variability. Those in favour of the fixed effect approach argue that the random effects model is using a statistical model to address a clinical problem. In particular, that it gives no insight into the source of between trial variability. Further, the trials are not a random sample – they are in fact all the trials that have been performed.

The chi-square test for statistical heterogeneity is not very powerful and will generally produce 'significant' results only when there is gross heterogeneity between the results of individual trials. As we have said, we expect clinical heterogeneity between trials, because of differing protocols, treatments, patients etc., and this will be true irrespective of whether the statistical test for heterogeneity is significant. If statistical heterogeneity is observed, then this should be the basis for investigating the possible explanations (Thompson, 1994). For example, perhaps the treatments are sufficiently different and can be split into less heterogeneous groups; perhaps dose or duration of treatment is the source of the heterogeneity; or perhaps it has something to do with the characteristics of the patients in the different trials. If a source of heterogeneity is found, the trials can be subdivided according to the appropriate characteristic and separate analyses can be made. In particular, if there is such heterogeneity, we may be able to approach answering questions such as which treatments perform best or which type of patients will benefit most from a particular treatment. Modelling such heterogeneity using the random effects approach is effectively throwing away valuable information.

In the search for the clinical source of statistical heterogeneity, there is likely to be some *post hoc* reasoning in the explanation, so that only cautious conclusions should be drawn after such data-delving. Nevertheless, the aim should be to minimise statistical heterogeneity within a comparison, so that it becomes almost irrelevant which model is used. Heterogeneity can be investigated fully only when data on individual patients are available, particularly when the end point is the time to an event. It is unlikely that in a literature-based meta-analysis all reasonable possible sources of heterogeneity can be investigated.

**Interpretation**

The patients included in randomised clinical trials are inevitably a selected subpopulation (Begg and Engstrom, 1987) of those patients with the disease and the true treatment effect is likely to vary in different situations, for example, with different types of patients. Thus, when extrapolating the results of a meta-analysis to different types of patients, biological reasons for possible differences both in size and direction of effect need to be considered. However, unless there is good evidence for differences of effect in different subgroups within the meta-analysis, the overall average estimate available is the best summary of the results of these trials, and because of the clinical heterogeneity of the trials, it is also perhaps the best estimate of the effect in the general population. The overall results of a meta-analysis therefore provide the best summary on the main end points from which each clinician, together with the patient, can assess the relative merits of different treatments.

The issue of what benefit is clinically worthwhile is clearly a subjective decision, likely to be influenced by factors such as the country, speciality of the clinician, patient-specific factors such as age and stage of disease, quality of life considerations and cost of the new treatment. For example, it is quite likely because of cultural differences that clinicians in the USA would accept a much smaller benefit to use toxic chemotherapy than clinicians in the UK (Parmar *et al*., 1996). It is also likely that oncologist may accept smaller benefits as an indication to offer chemotherapy than, for example, their surgical colleagues. In some countries the cost of some new therapies may be so prohibitive that it is impossible to give a new treatment unless the observed effect is enormous, which is very unlikely.

An important consideration must also be the appropriate interpretation of any survival benefit. For a number of good reasons survival differences between treatments are measured on a relative (the hazard ratio) scale. However, to aid interpretation they should also be presented on the absolute scale (Bobbio *et al*., 1994). For example, considering the complete results of the adjuvant use of CMF (Early Breast Cancer Trialists' Collaborative Group, 1992*a*,*b*), the estimated improvement with polychemotherapy in women below the age of 50 was a reduction in the relative risk of death by 25% (hazard ratio = 0.75). Table I relates this relative reduction in risk to an absolute improvement in survival given different underlying prognoses. One interpretation of this table could be that chemotherapy may be worthwhile for women with a poor or medium underlying risk of death, but that it may not be considered worthwhile for those with a very good prognosis.

The positive result of adjuvant CMF chemotherapy in women with early breast cancer highlights some of the differences in interpretation. When the results were first made available in the mid 1980s (Early Breast Cancer Trialists' Collaborative Group, 1988) there were a number of opinions of its interpretations and relevance around the world. For many clinicians in the US the results served to confirm their current treatment policy of routinely offering CMF chemotherapy to many of their patients. In Italy, where the CMF regimen was piloted, the results came as no great surprise, again confirming the policy of routinely offering CMF chemotherapy. In the UK, however, many clinicians were still sceptical about the benefit. The overall relative effect of CMF translated into an absolute survival benefit at 5 years across all stages of disease of 3.5% for all ages and 7% for premenopausal women. These modest benefits had to be balanced against the fact that CMF is a regimen that is relatively toxic for up to a year. After the updated results published in 1992 (adapted and presented in Figure 1), however, there appeared to be a greater acceptance of the role of CMF. This may be owing to a number of factors, perhaps including 10 year survival advantages and more conclusive results. The introduction of new and more effective anti-emetic regimens over this period may also have helped. Finally, the perception of a worthwhile effect may have changed, with findings emerging that patients were willing to accept fairly toxic regimens for relatively modest survival benefits (Slevin *et al*., 1990).

In contrast to the above positive results for CMF in early breast cancer, the example of cisplatin *vs* carboplatin in advanced ovarian cancer shows no evidence of difference between these two regimens. The estimated overall hazard ratio was 1.06, slightly in favour of cisplatin, with a 95% confidence interval of 0.94 to 1.18. The results translate into an absolute difference in 2 year survival of 2% in favour of cisplatin, with a 95% confidence interval of −3% to 6%. These results offer no good evidence that cisplatin is either superior or inferior to carboplatin and imply that if there is a difference between them it is very small. Comparing the two subtotals in Figure 2 also shows that there is no evidence of difference when cisplatin is replaced by carboplatin in combination regimens or as a single agent. Finally, it should be noted that no individual study stood out as indicating a significant benefit for either treatment.

This example illustrates the interpretation of an overall result of 'no difference' from a meta-analysis. In such situations, the onus lies on those championing the new treatment to convince the community at large that there is something special (producing both significant clinical and statistical heterogeneity) about an individual trial or trials

**Table I** Relationship between a hazard ratio of 0.75 (reduction in the relative risk of death of 25%) and absolute improvements over different underlying survivals[a]

| *Underlying (%)* | *Absolute improvements in survival (%)* |
|---|---|
| 30 | 11 |
| 40 | 10 |
| 50 | 9 |
| 60 | 8 |
| 70 | 6 |
| 80 | 5 |
| 90 | 2 |

[a]All calculations assume proportional hazards.

with purports to show a benefit. In doing this we must be aware of the possibility of extracting data-dependent results and choosing the most extreme trials to make a point. By contrast, substantial uncertainty may remain after a null result because the confidence interval is wide and worthwhile effects cannot be ruled out. In this case further large prospective trials randomised may be required before the issue is resolved.

### Conclusion

Often a number of randomised clinical trials which address the same or similar question are performed. There is a scientific and ethical obligation to summarise and present this information in an objective and quantitative manner. It will usually be possible to summarise the overall results on a few main end points in the form of a meta-analysis. Such a summary provides the firmest basis on which to assess the relative merits of competing therapies on end points such as survival or disease-free survival. To ensure an unbiased and complete summary it may be necessary to collect individual patient data from every relevant trial. In some cases a meta-analysis using data extracted from published reports may be sufficient and it certainly is a useful first step given the length of time it can take to collect individual patient data. Whichever approach is adopted it should be noted that a meta-analysis provides only a summary of the effect of treatments on *some* main end points and that neither individual trials nor meta-analysis provide prescriptions for how individual patients should be treated. Nevertheless, the clinical heterogeneity in a meta-analysis is likely to mean that the results are of more practical value than those of any individual trial. Issues such as the clinical relevance, appropriate extrapolation to individual patients, toxicity and implications on quality of life and cost of the treatment cannot easily be addressed within the meta-analysis. Currently, these have to be assessed in detailed local, regional or perhaps national studies. In this sense the meta-analysis acts only as one, although essential, source of information.

### References

ADVANCED OVARIAN CANCER TRIALISTS' GROUP. (1991). Chemotherapy in advanced ovarian cancer: an overview of randomised clinical trials. *Br. Med. J.*, **303,** 884–893.

BEGG CM AND ENGSTROM PF. (1987). Eligibility and extrapolation in cancer clinical trials. *J. Clin. Oncol.*, **5,** 962–968.

BOBBIO M, DEMICHELIS B AND GIUSTETTO G. (1994). Completeness of reporting trials' results: effect on physicians' willingness to prescribe. *Lancet*, **343,** 1209–1211.

BONNADONA G, BRUSAMOLINO E, VALAGUSSA P, ROSSI A, BRUGNATELLI L, BRAMBILLA C, DE LENA M, TANCINI G, BAJETTA E, MUSUMECI R AND VERONESI U. (1976). Combination chemotherapy as an adjuvant treatment in operable breast cancer. *N. Engl. J. Med.*, **294,** 403–411.

DERSIMONIAN R AND LAIRD N. (1986). Meta-analysis in clinical trials. *Controlled Clin. Trials*, **7,** 177–188.

DICKERSIN K, SCHERER R AND LEFEBVRE C. (1994). Identification of relevant studies for systematic review. *Br. Med. J.*, **309,** 1286–1291.

EARLY BREAST CANCER TRIALISTS' COLLABORATIVE GROUP. (1988). Effects of adjuvant tamoxifen and of cytotoxic therapy on mortality in early breast cancer. *N. Engl. J. Med.*, **319,** 1681–1691.

EARLY BREAST CANCER TRIALISTS' COLLABORATIVE GROUP. (1992*a*). Systemic treatment of early breast cancer by hormonal, cytotoxic or immune therapy. *Lancet*, **339,** 1–15.

EARLY BREAST CANCER TRIALISTS' COLLABORATIVE GROUP. (1992*b*). Systemic treatment of early breast cancer by hormonal, cytotoxic or immune therapy. *Lancet*, **339,** 72–85.

GOTZSCHE PC. (1987). Reference bias in reports of drug trials. *Br. Med. J.*, **295,** 654–656.

GRAY R, JAMES R, MOSSMAN J AND STENNING S ON BEHALF OF THE UK COORDINATING COMMITTEE ON CANCER RESEARCH (UKCCCR) COLORECTAL CANCER SUBCOMMITTEE. (1991). AXIS – A suitable case for treatment. *Br. J. Cancer*, **63,** 841–845.

GREGORY WM, RICHARDS MA AND MALPAS JS. (1992). Combination chemotherapy versus melphalan and prednisolone in the treatment of multiple myeloma: an overview of published trials. *J. Clin. Oncol.*, **10,** 334–342.

HIMMEL HN, LIBERATI A, GELBER RD AND CHALMERS TC. (1986). Adjuvant chemotherapy for breast cancer: a pooled estimate based on published randomised controlled trials. *J. Am. Med. Assoc.*, **256,** 1148–1159.

KING JF, GRANT A, KEIRSE MJNC AND CHALMERS I. (1988). Betamimetics in preterm labour: an overview of the randomised controlled trials. *Br. J. Obstet. Gynaecol.*, **95,** 211–222.

PARMAR MKB AND MACHIN D. (1995). *Survival Analysis: a Practical Approach.* John Wiley & Sons: Chichester.

PARMAR MKB, UNGERLEIDER R AND SIMON R. (1996). Why and when do we need confirmatory clinical trials? *J. Natl Cancer Inst.*, (submitted).

PETO R. (1987). Why do we need systematic overviews of randomised trials? *Stat. Med.*, **6,** 233–240.

PIGNON J, ARRIAGADA R, INDE DC, JOHNSON DH, PERRY MC, SOUHAMI RL, BRODIN O, JOSS RA, KIES MS, LEBEAU B, ONOSHI T, OSTERLIND K, TATTERSALL M AND WAGNER H. (1992). A meta-analysis of thoracic radiotherapy for small cell lung cancer. *N. Engl. J. Med.*, **327,** 1618–1624.

SIMON R. (1987). The role of overviews in cancer therapeutics. *Stat. Med.*, **6,** 389–396.

SIMON R AND ALTMAN DG. (1994). Statistical aspects of prognostic factor studies in oncology. *Br. J. Cancer*, **69,** 979–985.

SLEVIN ML, STUBBS L, PLANT HJ, WILSON P, GREGORY WM, JOANNE AMES P AND DOWNER SM. (1990). Attitudes to chemotherapy: comparing views of patients with cancer with those of doctors, nurses and general public. *Br. Med. J.*, **300,** 1458–1460.

STEWART LS AND CLARKE MJ ON BEHALF OF THE COCHRANE WORKING GROUP ON INDIVIDUAL PATIENT DATA. (1995). Practical methodology of meta-analyses (overviews) using individual patient data. *Stat. Med.*, **14,** 2057–2080.

STEWART LS AND PARMAR MKB. (1993). Meta-analysis of the literature or of individual patient data: is there a difference? *Lancet*, **341,** 418–422.

THOMPSON S. (1994). Why sources of heterogeneity in meta-analysis should be investigated. *Br. Med. J.*, **309,** 1351–1355.

WILTSHAW E, EVANS B AND HARLAND S. (1985). Phase III randomised trial cisplatin *vs* JM8 (carboplatin) in 112 ovarian cancer patients, stages III and IV. *Proc. Am. Soc. Clin. Oncol.*, **4,** 121.